# Roles of Mitochondrial DNA Damage in Kidney Diseases: A New Biomarker

**DOI:** 10.3390/ijms232315166

**Published:** 2022-12-02

**Authors:** Jun Feng, Zhaowei Chen, Wei Liang, Zhongping Wei, Guohua Ding

**Affiliations:** 1 Division of Nephrology, Renmin Hospital of Wuhan University, Wuhan 430060, China; 2 Nephrology and Urology Research Institute of Wuhan University, Wuhan 430060, China

**Keywords:** mitochondrial DNA, kidney diseases, mtDNA replication, mtDNA mutation, mtDNA leakage, mtDNA methylation

## Abstract

The kidney is a mitochondria-rich organ, and kidney diseases are recognized as mitochondria-related pathologies. Intact mitochondrial DNA (mtDNA) maintains normal mitochondrial function. Mitochondrial dysfunction caused by mtDNA damage, including impaired mtDNA replication, mtDNA mutation, mtDNA leakage, and mtDNA methylation, is involved in the progression of kidney diseases. Herein, we review the roles of mtDNA damage in different setting of kidney diseases, including acute kidney injury (AKI) and chronic kidney disease (CKD). In a variety of kidney diseases, mtDNA damage is closely associated with loss of kidney function. The level of mtDNA in peripheral serum and urine also reflects the status of kidney injury. Alleviating mtDNA damage can promote the recovery of mitochondrial function by exogenous drug treatment and thus reduce kidney injury. In short, we conclude that mtDNA damage may serve as a novel biomarker for assessing kidney injury in different causes of renal dysfunction, which provides a new theoretical basis for mtDNA-targeted intervention as a therapeutic option for kidney diseases.

## 1. Introduction

The kidney removes waste metabolic products through the glomerular filtration barrier and maintains water–electrolyte balance via renal tubular reabsorption. As an organ with high energy demand, the kidney has abundant mitochondria to generate ATP to sustain its internal homeostasis. An increasing number of studies have revealed that mitochondrial dysfunction plays a vital role in the occurrence and progression of kidney diseases, including acute kidney injury (AKI) and chronic kidney disease (CKD) [1,2]. However, the mechanism of mitochondrial dysfunction remains undefined.

Mitochondrial DNA (mtDNA) is a double-stranded circular DNA that is independent of nuclear DNA. Normally, mtDNA with a length of 16,596 base pairs is located in the mitochondrial matrix. mtDNA possesses its own transcriptional and translational system and encodes 2 rRNAs, 22 tRNAs, and 13 polypeptides. These polypeptides include ND1-6, ND4L, COXⅠ-Ⅲ, cyt-b, ATPase6, and ATPase8, which are involved in the composition of the mitochondrial respiratory complexes to maintain the integrity of the electron transport chain (ETC) and the stability of oxidative phosphorylation (OXPHOS) [3]. The functioning of OXPHOS provides physiologically required energy to cells and organs, and the ETC is the main source of reactive oxygen species (ROS) production. mtDNA damage leads to inefficient mitochondrial function, such as ROS overproduction, decreased ATP generation and altered metabolite profiles [4]. Damaged mtDNA was accompanied by oxidative stress activation and reduced mitochondrial mass [5]. Importantly, mtDNA integrity is closely related to mitochondrial function. In addition to determining mitochondrial function directly, mtDNA can also act as an endogenous pathogenic factor. Curiously, the latest studies have reported that mtDNA leaking into the cytoplasm can act as an inflammatory mediator and activate the natural immune inflammatory response [6,7].

The process of DNA transcription and replication is often accompanied by mutations, deletions, insertions, translocations and strand breaks. These damages could be rapidly reversed by the nuclear DNA repair system to promote genomic integrity [8]. However, mtDNA is absent of mature damage-sensing signaling and protective histones. Excessive ROS production mediates oxidative stress damage in cells and can also exacerbate mtDNA mutations, strand breaks and deletions, creating a vicious cycle of mtDNA damage [9]. Therefore, it is more sensitive to be impaired by internal and external unfavorable factors.

**Figure 1:**
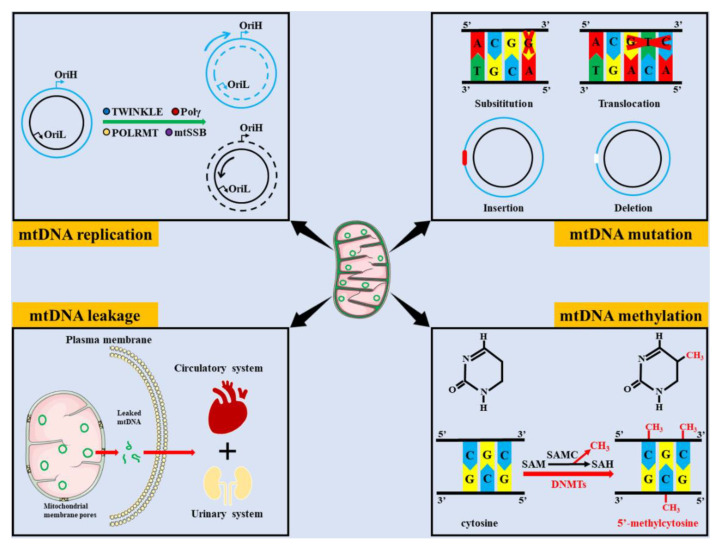
Common types of mtDNA damage. mtDNA damage includes impaired mtDNA replication, mtDNA mutations, mtDNA leakage and mtDNA methylation. mtDNA replication operates in a semi-conserved manner and contains multiple enzymes, such as TWINKLE, Pol γ, POLRMT, and mtSSB, which may hinder mtDNA replication when they are disrupted. The common types of mtDNA mutation includes substitution, translocation, insertion, and deletion. Leaked mtDNA can be transferred to peripheral plasma and urine via the circulatory and urinary systems, respectively. Under the action of DNMTs, the methyl donor compounds derived from SAM are transferred to CpG islands to form 5′-methylcytosine. (OriH, the origin of heavy strand replication; OriL, the origin of light strand replication; Pol γ, polymerase gamma; POLRMT, mitochondrial RNA polymerase; mtSSB, mitochondrial single-strand binding protein; SAM, S-adenosyl-L-methionine; SAH, S-adenosyl-L-homocysteine; SAMC, S-adenosylmethionine carrier; DNMTs, and DNA methyltransferases).

The roles of mtDNA damage in multiple diseases, such as cancers, cardiovascular diseases, liver diseases, and neurological diseases, have attracted much attention [10,11,12,13]. Similarly, it has been reported that mtDNA damage, including impaired mtDNA replication, mtDNA mutations, mtDNA leakage, and mtDNA modification, plays an important role in the progression of kidney diseases (Figure 1). In this review, we summarize the roles of mtDNA damage in kidney diseases and highlight the potential value for diagnostic and therapeutic targets.

## 2. Common Types of mtDNA Damage

### 2.1. Impaired mtDNA Replication

The stability of mtDNA is essential for maintaining a healthy function of mitochondria within cells. mtDNA replication and distribution within mitochondrial networks play an important role in sustaining mitochondrial homeostasis. Similar to nuclear DNA replication, mtDNA replication operates in a semiconserved manner and entails multiple types of mechanisms, including strand-displacement and strand-coupled models [14]. In each cell cycle, mtDNA replicates multiple times, and the replication of both strands, the heavy strand and light strand, is not synchronized. mtDNA replication is closely related to mitochondrial metabolism, and its activity could be affected by alterations in specific mitochondrial metabolites, such as nucleotides and NAD^+^. Interestingly, exogenous supplementation with the NAD^+^ precursor, beta-nicotinamide mononucleotide, increased the pool of mitochondrial nucleotides, and promoted mtDNA replication [15]. Decreased mtDNA copy number is associated with increased oxidative stress through ROS overproduction, which further results in mitochondria-related metabolic disorders and apoptosis [16].

A variety of related enzymes and regulatory factors are involved in mtDNA replication, such as mtDNA polymerase γ (POL γ), mitochondrial single-strand binding protein (mtSSB), mitochondrial helicase TWINKLE, topoisomerase, and mitochondrial transcription factor A (TFAM) [17,18]. All of these factors are mainly encoded by nuclear genes. Therefore, mtDNA replication is regulated by both its own and nuclear DNA. POL γ-deficient cells experience severe deletion of mtDNA, which is rescued by enhancing mitochondrial deoxyribonucleoside triphosphate production [19]. Activating transcription factor associated with stress 1 (ATFS-1) is absent in healthy mitochondria owing to its degradation by the mtDNA-bound protease Lon peptidase 1 (LONP-1), but it accumulates in damaged mitochondria. LONP-1 inhibition increases ATFS-1 and PLO γ binding to mtDNA and promotes mtDNA replication thereby improving the mtDNA heteroplasmy ratio and restoring OXPHOS [20]. mtSSB is critically required for restricting transcription initiation to optimize RNA primer formation at two origins of mtDNA replication and its mutations affect mtDNA replication and induce mtDNA deletion [21,22]. TFAM deficiency aggravates reduction of mtDNA copy number and OXPHOS [23]. In summary, complete mtDNA replication is an orderly process. When any one of these steps is disrupted, it may cause impaired mtDNA replication.

### 2.2. mtDNA Mutations

mtDNA appears to be more frequent and susceptible to mutations than nuclear DNA. mtDNA mutations are commonly found in maternally inherited diseases, and unfavorable environmental factors can also cause sporadic mtDNA mutations [24]. Both the gene-coding region and displacement loop (D-loop), which plays a role in transcriptional regulation, can be mutated in mtDNA, thus affecting the vital regions of the genome.

Because each mitochondrion contains a different amount mtDNA copies, the types of mtDNA mutations can be divided into homogeneous and heterogeneous mutations. Homogeneous mutations refer to the mutation of all mtDNA in mitochondria, whereas heterogeneous mutations refer to the coexistence of mutant and wild-type mtDNA. mtDNA mutations have the cumulative effect of defective mitochondrial function, causing impairment of a cellular energy supply [25]. The biological consequences of mutations depend on the proportion of mutant mtDNA and the type of mutation carried by the cells. The minimum number of copies of mtDNA mutations that cause dysfunction in specific tissues and organs is called the threshold value, and the lower the threshold value, the greater is the probability of disease occurrence [26]. The mutation threshold value has important implications for the clinical manifestation of energy dependence and the disease and the threshold value are different between individual tissues and organs.

With the continuous improvement of detection technologies, mtDNA mutation-related diseases are being discovered and increasingly gain the attention of scholars and clinicians. Studies have found that mtDNA mutations are associated with the development of numerous diseases, including cardiovascular diseases, kidney diseases, tumors, and aging [27,28,29,30]. Currently, there is a lack of effective treatment for mtDNA mutation-related diseases, which mainly focuses on improving clinical symptoms. Therefore, it is particularly important to accurately detect the sites of mtDNA mutations.

### 2.3. mtDNA Leakage

**Figure 2:**
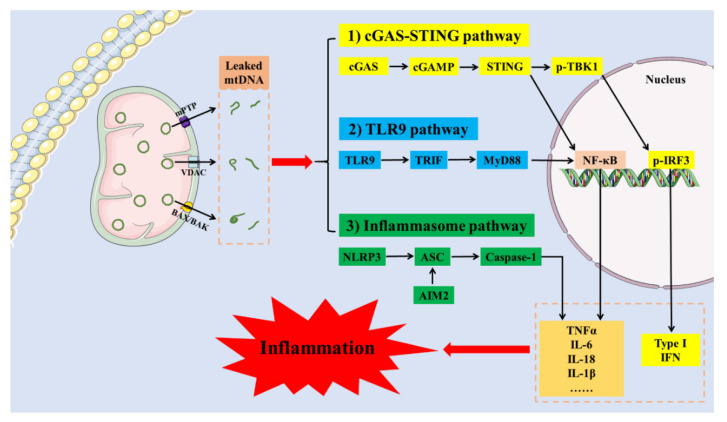
mtDNA leakage induces inflammation activation. mtDNA is released into the cytoplasm through several pathways, such as the BAK/BAX pore, VDAC oligomer pore, and mPTP. mtDNA released into the cytoplasm activates inflammatory responses through multiple signaling pathways, including cGAS-STING, TLR9, NLRP3, and AIM2 inflammasome. (cGAS, cyclic GMP-AMP synthase; STING, stimulator of interferon genes; cGAMP, cyclic guanosine monophosphate-adenosine monophosphate; p-TBK1, phospho-TANK-binding kinase-1; TLR9, toll-like receptor 9; TRIF, TIR domain-containing adapter-inducing IFN β; MyD88, myeloid differentiation protein 88; NLRP3, nod-like receptor pyrin 3; ASC, apoptosis associated speck like protein; AIM2, absent in melanoma 2; VDAC, voltage-dependent anion channel; mPTP, mitochondrial permeability transition pore; TNFα, tumor necrosis factor α; IL-6, interleukin-6; IL-18, interleukin-18; IL-1β, interleukin-1β; NF-κB, nuclear factor kappa-B; p-IRF3, phospho-interferon regulatory factor-3; IFN, interferon).

The mitochondrial membrane is similar to the cell membrane and has a bilayer structure to maintain the integrity of mitochondria. Lipids and proteins are the main components of the inner mitochondrial membrane (IMM) and outer mitochondrial membrane (OMM). The difference in their composition determines that the IMM and OMM have different physiological functions. The OMM contains a large number of integral membrane proteins that regulates mitochondrial permeability. In contrast, the IMM is composed of a variety of carrier proteins and metabolism-related enzymes that are responsible for complex mitochondrial biochemical reactions. Normally, mtDNA is encapsulated in the mitochondrial matrix. Once the structural integrity of mitochondrial membrane is disrupted, mtDNA is released into the cytoplasm. Defective mitochondrial membrane structure and increased permeability are key causes of mtDNA leakage. Altered lipid composition in the mitochondrial membrane leads to increased mitochondrial permeability and mtDNA leakage [31]. Mitochondrial damage caused by multiple factors is often accompanied by mtDNA leakage. Recent studies revealed that mtDNA can be released into the cytoplasm through several pathways, such as the BAK/BAX pore, voltage-dependent anion channel (VDAC) oligomer pore and mitochondrial permeability transition pore (mPTP) [32,33,34], as shown in Figure 2. However, these processes have been studied to an inconsistent extent among different diseases.

Accumulated mtDNA in the cytoplasm is recognized as an endogenous pathogen and activates innate immune and inflammatory responses [12,35]. mtDNA released into the cytoplasm can activate inflammatory responses through multiple signaling pathways, including the cyclic GMP-AMP synthase (cGAS)-stimulator of interferon genes (STING), toll-like receptor 9 (TLR9), nucleotide-binding oligomerization domain-like receptor protein 3 (NLRP3) and absent in melanoma (AIM2) inflammasome signaling pathways [36]. cGAS is a member of the nucleotidyl transferase family and contains a DNA-binding region, which recognizes endogenous and exogenous DNA, including viral DNA, mtDNA, chromosomal terminal telomeric repeat sequence DNA, and cytoplasmic chromatin fragments. The recognition of DNA by cGAS is length-dependent, and DNA can effectively bind to cGAS when the length of dsDNA is >45 bp [37]. The phosphoribose backbone of DNA binds to cGAS in a non-sequence-dependent manner and induces conformational changes [38]. mtDNA released into the cytoplasm or peripheral circulation is recognized by cGAS and catalyzes the generation of the second messenger cyclic guanosine monophosphate-adenosine monophosphate (cGAMP), which further activates STING-related pathways, including the type 1 interferon (IFN) response and the classical NF-κB inflammatory pathway, thereby activating the immune inflammation [39]. TLR9 is a transmembrane protein mainly located in the endoplasmic reticulum, which contains an extracellular region that recognizes pathogen-associated molecular patterns (PAMPs) and an intracellular region including a toll/interleukin-1 receptor (TIR) structure for downstream signaling. TLR9 can act as a DNA recognition receptor and participate in the natural immune response in human body. A growing number of studies suggest that TLR9 plays an important role in the development of autoimmune diseases [40,41]. TLR9 is a member of the TLR family of proteins that are most closely related to mtDNA [42]. mtDNA activates TLR9 and is involved in cytokine production, splenic apoptosis and renal injury in sepsis [43]. TLR9 mediates the formation of inflammatory responses by activating the NF-κB inflammatory pathway via myeloid differentiation factor 88 (MyD88) [44]. NLRP3 activates caspase-1 and gasdermin D (GSDMD), releasing large amounts of inflammatory factors and initiating pyroptosis, a new orderly mode of cell death [45]. NLRP3 can recognize both PAMPs and danger-associated molecular patterns (DAMPs), and activate NLRP3 inflammasome, which consists of NLRP3 receptor protein, apoptosis-associated speck-like protein (ASC) and caspase-1 precursor protein (pro-caspase-1). A recent study found that mtDNA leaking into the cytoplasm activated NLRP3 inflammasome in brown adipose tissue, which is engaged in obesity-induced insulin resistance and impaired thermogenesis [46]. The double-stranded DNA receptor AIM2 also recognizes mtDNA released through BAK/BAX pores, and triggers IL-1β secretion and pyroptosis [47].

### 2.4. mtDNA Methylation

DNA methylation is one of the most widely studied epigenetic mechanisms. Under the action of DNA methyltransferases (DNMTs), the methyl donor compounds derived from S-adenosylmethionine (SAM) are transferred to CpG islands to form 5′-methylcytosine (5′-mC), which is the most common type of DNA methylation. DMNTs include two major groups, DNMT3a and DNMT3b, which catalyze the de novo methylation of unmethylated DNA duplexes, and DNMT1, which maintains the methylation state of DNA after semiconserved replication. It is reported that DNMTs plays a significant role in regulating tricarboxylic acid metabolites, mitochondrial respiration and oxidative stress [48,49].

Recent studies have revealed that mtDNA can also be methylated and methylated mtDNA underlies disease progression [50]. The levels of mtDNA methylation may be influenced by a number of intracellular or extracellular factors. Significant abnormalities in mtDNA methylation occur in a variety of diseases, particularly in mitochondria-related diseases [51]. Methylation of mitochondrial genome could lead to the etiology of human disease and altered methylation levels of mtDNA were assessed in animal models and human tissue from patients with obesity, diabetes, cancer, and cardiovascular and neurodegenerative diseases [14]. Hypermethylation of mtDNA genes also induces systemic insulin resistance and a related metabolic disorder [52].

mtDNA methylation is an emerging and incompletely understood phenomenon that regulates mitochondrial function. mtDNA methylation drift at different sites of coding gene loci results in decreased mtDNA copy number and altered gene expression [53]. The degree of mtDNA methylation was negatively correlated with mtDNA content [54]. mtDNA methylation can be considered as an early molecular event and a potential biomarker for effective disease prediction and diagnosis. mtDNA methylation is a reversible epistatic modification, making it an important therapeutic target. Gene editing of mtDNA methylation is still in the basic research and early clinical stage and, as such, safety issues cannot be ignored. It is hoped that with the development of mitochondrial gene editing technology, it will help us to further understand how mtDNA is methylated.

## 3. mtDNA Distribution in Kidney Diseases

**Figure 3:**
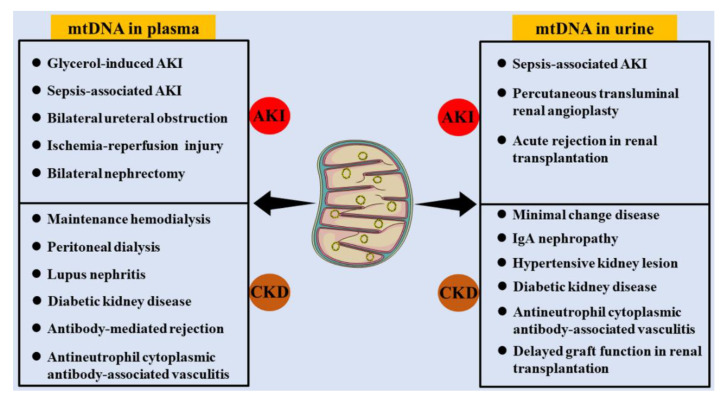
mtDNA distribution in kidney diseases. mtDNA can be detected in both peripheral plasma and urine of multiple kidney diseases including AKI and CKD. (AKI, acute kidney injury; CKD, chronic kidney disease; IgA, Immunoglobulin A).

Generally, intact mtDNA is present in the mitochondrial matrix but not in the cellular matrix, peripheral blood, or urine. However, mitochondrial damage contributes to cell injury in multiple diseases and is often followed by mtDNA leakage from mitochondria. When leaked mtDNA are insufficient to clean by cellular repair and phagocytosis system, they could be released into the peripheral circulation. mtDNA in the peripheral circulation filtrates through the glomerular filtration barrier and participates in the formation of urine. Cell detachment in the urinary system, such as the bladder and ureter, can also lead to the presence of mtDNA in the urine. Therefore, mtDNA can be detected in both peripheral plasma and urine. The levels of mtDNA in peripheral blood and urine can be used to assess mitochondrial function and the status of some organs. There is also an increasing number of studies on the correlations of mtDNA distribution and kidney function (Figure 3).

### 3.1. mtDNA in Peripheral Serum

The levels of mtDNA in peripheral serum is relatively low under normal physiological conditions, and its concentration is increased with damage to several organs or tissues, such as the kidney, heart, liver, brain, and muscle [55,56,57,58]. A correlation between plasma mtDNA and kidney diseases, including AKI and CKD, has been reported. In addition, plasma mtDNA has been considered as an indicator for assessing kidney injury.

Numerous factors can trigger the occurrence of AKI, including bilateral ureteral obstruction, sepsis-associated AKI, glycerol-induced AKI, ischemia-reperfusion injury (IRI) and bilateral nephrectomy [59]. The predictive role of plasma mtDNA in AKI has been taken into consideration. For example, the plasma mtDNA level was increased in AKI patients with sepsis [60]. In glycerol-induced AKI rats, the concentration of plasma mtDNA was increased after 3 h, indicating that plasma mtDNA could be an early and sensitive biomarker of AKI [61].

A later study in chronic renal insufficiency cohort reported that the lower mtDNA copy number was correlated with a higher risk of CKD progression, independent of established risk factors in CKD patients [62]. mtDNA release in platelets driven by a receptor for immune complex FcγRIIA is the key source of mitochondrial antigens in systemic lupus erythematosus [7]. By pumping excess mtDNA into the circulation of mice, a high level of serum mtDNA can trigger inflammation and induce kidney damage [63]. Plasma mtDNA is a strong predictor of cardiovascular events as well as the need for hospitalization in patients with peritoneal dialysis [64]. In patients undergoing maintenance hemodialysis (MHD), circulating mtDNA contents were significantly higher in sarcopenia patients, together with higher TLR9 and IL-6 expression, which demonstrated that mtDNA could be involved in the pathogenesis of MHD-related sarcopenia [65]. As one of the indispensable proteins encoded by mtDNA, serum ND6 was increased in active antineutrophil cytoplasmic antibody-associated vasculitis, and ND6 concentration was negatively correlated with the percentage of normal glomeruli in kidney biopsies [66]. These studies revealed that serum mtDNA reflected immune inflammatory status and kidney injury.

Serum mtDNA-mediated immune rejection determines the efficacy of renal transplantation. The higher the level of serum mtDNA in the kidney donor, the more likely kidney transplant recipient is to experience antibody-mediated rejection. Therefore, donor serum mtDNA can be used as a predictive marker for antibody-mediated rejection and for validated donor organ evaluation [67]. Accordingly, donor plasma mtDNA was an independent risk factor for delayed graft function (DGF) of renal recipient, which was valuable in organ evaluation [68].

### 3.2. mtDNA in Urine

mtDNA in urine can be used as an indicator to assess kidney function. An acute rise in urinary mtDNA by percutaneous transluminal renal angioplasty reflects renal mitochondrial damage and thus inhibits renal recovery [69]. In patients with sepsis, elevated urinary mtDNA levels were associated with mitochondrial dysfunction and renal injury, suggesting that sepsis causes renal mitochondrial injury. Therefore, urinary mtDNA may be considered a valuable biomarker for determining the development of AKI and mitochondria-targeted therapy following sepsis-induced AKI [70]. Compared to healthy controls, the expression of STING in kidney was increased and urinary mtDNA levels were elevated in minimal change disease (MCD) patients, which could be used as a valuable prognostic marker in MCD [71]. Urinary mtDNA levels were significantly elevated in both diabetic patients and mice, which was negatively correlated with glomerular filtration rate and positively correlated with interstitial fibrosis [63,72]. mtDNA was also readily detected in the urinary supernatant of nondiabetic CKD and its level correlated with the rate of decline in renal function and predicted the risk of elevated serum creatinine and the need for dialysis in CKD patients [73]. Low urinary mtDNA was significantly correlated with favorable renal outcomes at 6 months follow-up, indicating the novel prognostic role of mtDNA for renal outcome in CKD patients [74]. In renovascular hypertensive patients, elevated urinary mtDNA copy numbers were correlated with mitochondrial dysfunction and renal injury, including increased urinary neutrophil gelatinase-associated lipocalin, kidney injury molecular-1 (KIM-1) levels and decreased estimated glomerular filtration [75,76]. Higher urinary mtDNA copy numbers and higher mean annual rates of estimated glomerular filtration rate (eGFR) decline were displayed in minor glomerular abnormalities and IgA nephropathy (IgAN) patients, and mitochondrial injury could be prior to pathological changes and increased proteinuria [77,78]. Urinary mtDNA was elevated in antineutrophil cytoplasmic autoantibodies-associated vasculitis (ANCA-AAV) patients suffering from abnormal kidney function and its level correlated with the severity of renal injury and pathological neutrophil infiltration [79]. Urinary mtDNA level was corelated with cold ischemia time and renal function in human renal transplant recipients, which was associated with renal allograft function and the diagnosis of DGF following renal transplantation [80]. The urinary mtDNA level was significantly higher in patients with acute rejection and DGF, which could be predictive of short-term post-transplant renal function [81]. Taken together, the above studies demonstrate that mtDNA in urine is closely associated with alterations in renal function in a variety of kidney diseases and a high level of urinary mtDNA is an unfavorable factor.

## 4. mtDNA Damage in Kidney Diseases

### 4.1. Impaired mtDNA Replication

mtDNA replicates in cultured cells from kidney through the asynchronous mechanism [82]. Decreased mtDNA copy number in blood specimens was associated with abnormal levels of serum creatinine, which indicated impaired kidney function [83]. During mtDNA replication, the role of mtSSB1 is to protect displaced single-stranded DNA from damage, to prevent the formation of DNA secondary structures and the binding of inappropriate DNA synthesis and catabolic enzymes. Gustafson et al. reported a case of young CKD patient harboring a mtSSB1 mutation (p.E27K) accompanied by a single large-scale mtDNA deletion [84]. mtDNA content of the kidney was also significantly decreased in SSBP1 mutation (p.R107Q) patients who displayed impaired OXPHOS and kidney function insufficiency requiring transplantation [85]. Except for mtDNA of brain compartments, kidney mtDNA was the most vulnerable to accumulation of age-related damage and the copy number of mtDNA in kidney of aged rats was significantly increased [86]. Furthermore, mtDNA levels were significantly higher in the proximal and distal tubules than in the glomerular and collecting duct epithelium of kidney. With increasing age, mtDNA content was decreased in renal tubules, which was consistent with the gradual decline of kidney function and could be reversed by caloric restriction [87]. Overall, mtDNA replication is a biomarker of mitochondrial function, which is associated with increased mortality and morbidity in age-related diseases [88].

Recent studies have revealed that impaired mtDNA replication contributed to AKI. mtDNA replication and content were decreased with enhanced mitophagy in kidney, which contributed to the occurrence of AKI and increased mortality in liver transplantation rats [89]. mtDNA copy number was decreased in renal fibrosis models, including unilateral ureteral obstruction (UUO) and IRI, accompanied with mitochondrial dysfunction and oxidative stress [90]. Hypoxia inducible factor-1α (HIF-1α)-BCL2/adenovirus E1B 19 kDa protein-interacting protein 3 (BNIP3)-mediated mitophagy regulated mtDNA copy number and ROS production and inhibited cell apoptosis in renal tubular cells of IRI model [91].

Various causes of CKD exist with abnormal mtDNA replication. The reduced mtDNA copy number in peripheral blood mononuclear cells of MHD patients predicted poor clinical outcomes [92]. Consistently, mtDNA copy number was decreased in kidneys of diabetic mice, accompanied by downregulated TFAM expression and ATP production [93]. Similarly, our previous study demonstrated that impaired mtNDA replication in podocytes contributed to kidney injury in diabetic kidney disease (DKD) [94]. mtDNA deletion also aggravated podocyte injury and depletion, which was involved in the pathogenesis of focal segmental glomerulosclerosis (FSGS) [95]. A decrease in mtDNA content was the main cause of reduced OXPHOS in chromophobe renal cancer (ChRCC) [96]. The latest study showed that mtDNA replication defects led to the formation of mtDNA linear deletion, which triggered an immune response and led to progressive kidney disease in aging animals [97]. 

### 4.2. mtDNA Mutations

The kidney is not only an organ with high mtDNA replication. It also contains multiple mtDNA mutation sites that can be mutated [98]. The downstream effect of mtDNA mutations is mitochondrial dysfunction. mtDNA mutations usually cause systemic diseases, which may also be associated with the progression of AKI and CKD [28]. The kidney diseases caused by mtDNA mutations at different loci are summarized in Table 1.

An adenine to guanine substitution at nucleotide 3243 of the mtDNA (m.3243A>G), which affects the mitochondrial MT-TL1 gene, has been shown to cause mitochondrial encephalomyopathy, lactic acidosis, and stroke-like episodes (MELAS) syndrome. The most striking characteristics of renal biopsy were FSGS and arteriolar hyaline thickening [116]. Cai et al. recently reported that a patient with m.3243A>G mutation was diagnosed with membranous nephropathy, and AKI complicated by hyperuricemia might be attributed to mtDNA mutations [99]. 

A novel heteroplasmic nonsense mtDNA mutation m.6145G>A in the mitochondrial cytochrome c oxidase subunit I (MTCO1) was also identified in a patient who exhibited mitochondrial abnormalities, chronic tubulointerstitial changes and recurrent episodes of rhabdomyolysis [100]. Mitochondrial tubulointerstitial nephropathy (MITKD) is a tubulointerstitial nephropathy caused by mutations in mtDNA. m.616T>C is one of the mutations that lead to MITKD, the main symptoms of which are chronic renal insufficiency and epilepsia [101]. It has also been reported that two mt-ND5 pathogenic variants m.13513G>A and m.13514A>G caused mitochondrial dysfunction in tubulo-interstitial kidney disease [102]. Aristolochic acid elevated the levels of mutagenic 8-oxo-2′-deoxyguanosine and 7-(deoxyadenosine-N6-yl)-aristolactam adduct on mtDNA isolated from human HEK293 cells, which shed light on a potentially important causative role of mtDNA mutations and mitochondrial dysfunction in the etiology of aristolochic acid nephropathy [117]. Patients with type 2 diabetes diagnosed with the m.4216T>C mtDNA mutation are more likely to have poor glycemic control, which triggers the progression of DKD [103]. However, an mtDNA mutation m.8344A>G in mitochondrial tRNALys gene causing myoclonic epilepsy with ragged-red fibers (MERRF) syndrome has no influence on mtDNA copy number and kidney function [118].

Comprehensive mtDNA resequencing has been applied for detecting mutations in clinical samples and several kinds of tumor tissues have been examined for mtDNA mutations, including that of renal cell carcinoma (RCC) [119]. Mutation analysis of mtDNA showed mutations in the gene of mitochondrial complex Ⅰ subunit ND1, ND5, and ND6, which contributes to respiratory chain deficiency and the occurrence of renal oncocytoma [104]. ChRCC is a subtype of RCC, which is accompanied by a high rate of mtDNA mutations [120]. mtDNA sequencing analysis revealed that mtDNA mutations led to function deficiency of NADH dehydrogenase subunits, which further promoted the transformation of the metabolic pattern in ChRCC [105].

### 4.3. mtDNA Leakage

The kidney is an organ rich in mtDNA. When the kidney is stimulated by harmful factors, mtDNA can be released from mitochondrial matrix to cytoplasm. Cisplatin induced mtDNA leakage into the cytosol, likely through BAX pores in the mitochondrial outer membrane in kidney tubules with subsequent activation of the cGAS-STING pathway, thereby triggering inflammation and AKI progression [121]. The receptor-interacting protein kinase 3 translocates to mitochondria and interacts with mitofilin, resulting in increased mtDNA release and activation of cGAS-STING-p65 pathway in renal IRI [122]. In mice with tubule-specific knockout of TFAM, aberrant packaging of mtDNA resulted in cytosolic translocation, which further activated the cytosolic cGAS-STING pathway and recruited cytokine and immune cells to aggravate kidney fibrosis [123]. In DKD, the downregulation of superoxide dismutase 2 mediated mitochondrial dysfunction and mtDNA leakage, which could activate TLR9 in macrophages [124]. In summary, mtDNA leakage may be a concomitant phenomenon of kidney injury and mediates the development of multiple inflammatory responses.

### 4.4. mtDNA Methylation

Current studies on mtDNA methylation in kidney are still a blind spot. Only a single study on the correlation between renal tumor metastasis and mtDNA methylation was reported. It was demonstrated that, compared to primary RCC cells, the D-loop region of mtDNA was markedly hypermethylated in bone metastatic RCC cells, which provided a direct link between hypermethylation of mtDNA in RCC and tumor growth in bone metastases [125]. Due to technology limitations and cost challenges in detecting mtDNA methylation, the study progress in this area is relatively poor. With sustained attention and dedication against investigation to this field, the role of mtDNA methylation in kidney diseases will be continuously explored.

## 5. Pharmacological Intervention of mtDNA Damage in Kidney Diseases

As discussed in the previous section, the content and integrity of mtDNA in the kidney is impaired in the etiology of a variety of kidney diseases. mtDNA damage can directly affect mitochondrial function. Therefore, targeted interventions against mtDNA damage may have a therapeutic effect on kidney diseases. 

A wide variety of agents are currently applied to intervene in renal mtDNA damage, as shown in Table 2. We summarized the pharmacological mechanisms of these agents, and found that most of them alleviate kidney injury and cellular apoptosis by improving mitochondrial functions, such as increased mtDNA content, peroxisome proliferator-activated receptor gamma coactivator-1α (PGC-1α) and peroxisome proliferator-activated receptor γ (PPAR γ) expression, which promote mitochondrial OXPHOS to facilitate energy supply and reduce oxidative stress, ROS production, and inflammation. There are also a few therapeutic agents, such as l-carnitine and sacubitril/valsartan, which attenuate the inflammatory response activated by mtDNA leakage via suppressing inflammation-related pathways, such as the TLR9 and cGAS-STING signaling pathways [124,126].

A number of other alternative therapies have also been reported. For instance, extracellular vesicles (EVs) derived from mesenchymal stem cells (MSCs) contain functional mitochondrial components, such as mtDNA, mitochondrial proteins, and energy-related proteins from the tricarboxylic acid cycle [135]. MSC-EV-mediated TFAM mRNA transfer restored TFAM expression, mtDNA deletion, and OXPHOS defects in renal tubular cells of AKI [136]. The latest research shows that mitochondrial transplantation might be a novel treatment for mitochondrial diseases. Direct exogenous supplementation of mitochondria can replace damaged mtDNA, restore mitochondrial function, and inhibit oxidative stress, thus reducing apoptosis [137,138]. Moreover, mitochondrial replacement therapy can be used for maternally inherited diseases caused by mutations in mtDNA [139].

## 6. Conclusions and Future Perspectives

Intact mtDNA is closely related to mitochondrial function. mtDNA lacks a sophisticated self-repair system and is susceptible to a variety of external and internal factors, including drugs, infections, immune system disorders, hypertension, diabetes, and aging. All these etiologies can lead to mtDNA damage, which further augments mitochondrial dysfunction, manifested as defective ETC, reduced OXPHOS, and oxidative stress and inflammatory response, thus participating in the process of kidney injury. Therefore, it is imperative to explore the role of mtDNA damage in kidney disease.

In this review, we have scrutinized the common types of mtDNA damage, including impaired mtDNA replication, mtDNA mutations, mtDNA leakage, and mtDNA methylation. The mechanisms of these damage types were comprehensively described, and the studies related to mtDNA damage in kidney diseases were also summarized in detail. It has been revealed that mtDNA damage plays an important role in kidney diseases and the levels of mtDNA in peripheral plasma and urine have indicated a marker role in kidney diseases. Pharmacological treatment or exogenous mtDNA transplantation can improve damaged mtDNA, restore mitochondrial function, or directly inhibit mtDNA-induced inflammatory response, thus providing a theoretical basis and new avenues for the treatment of kidney diseases. In conclusion, mtDNA damage serve as a key biomarker of kidney diseases.

## Figures and Tables

**Table 1:** mtDNA mutations in kidney diseases.

Mutation	Gene	Clinical Characters	Diagnosis	Reference
m.616T>C	MT-tRNA^Phe^	Recurrent swelling and pain	CKD	[28]
		Renal insufficiency	Hyperuricemia	
m.3243A>G	MT-TL1	Chest tightness and shortness of breath	Membranous nephropathy	[99]
		Hyperlactatemia		
		Hyperuricemia		
		Proteinuria		
m.6145G>A	MT-CO1	Chronic tubulointerstitial changes	Rhabdomyolysis	[100]
		Elevated serum creatinine level Dark-colored urine Mitochondrial abnormalities		
m.616T>C	MT-tRNA^Phe^	Developmental delay Epilepsy Hypertension Electrolyte disturbance Chronic renal insufficiency	Autosomal-dominant tubulointerstitial kidney disease	[101]
m.13513G>A m.13514G>A	MT-ND5	Anuric AKI	Tubulo-interstitial kidney disease	[102]
		Acute pulmonary edema		
		Hyperlactatemia with metabolic acidosis		
		Proteinuria		
		Hypertension		
m.4216T>C	MT-ND1	Higher levels of fasting glucose	DKD	[103]
		Decreased renal function		
3571_3572insC	MT-ND1	-	Renal oncocytoma	[104]
3571delC				
10952_10953insC	MT-ND4			
11038delA				
12384_12385insT	MT-ND5			
12390_12391insC				
m.13493T>C				
m.3243A>G	MT-TL1			
m.3565T>AC	MT-ND1	-	ChRCC	[105]
m.3922G>A				
m.4569G>A	MT-ND2			
m.4969G>C				
m.10806G>A	MT-ND4			
m.11866A>AC				
m.12384TC>T	MT-ND5			
m.12417C>CA				
m.13127AC>A				
m.13206CTG>C				
m.13230CA>C				
m.14159C>A	MT-ND6			
m.6490T>C	MT-CO1			
m.9651C>T	MT-CO3			
m.3243A>G	MT-TL1	Proteinuria Decreased eGFR Hyperuricemia	FSGS	[106]
			Nephrosclerosis	
			DKD	
			Tubulointerstitial nephropathy	
			Minor glomerular abnormality	
m.3243A>G	MT-TL1	Osteoporosis	Nephrolithiasis	[107]
		Bilateral sensorineural deafness		
		Sensory axonal neuropathy		
m.6129G>A	MT-CO1	-	Von Hippel-Lindau renal oncocytoma	[108]
m.8993T>G	MT-ATP6	Proteinuria Decreased eGFR	Neuropathy, ataxia and retinitis pigmentosa syndrome	[109]
			End-stage renal disease	
m.3243A>G	MT-TL1	Wolff-Parkinson-White syndrome Proteinuria	Chronic progressive external ophthalmoplegia	[110]
			FSGS	
m.547A>T	MT-HSP	Interstitial fibrosis	Tubulointerstitial kidney disease	[111]
m.616T>C	MT-tRNAPhe	Tubular atrophy		
m.09155A>G	MT-ATP6	Central obesity Proteinuria Impaired glucose tolerance	Maternally inherited deafness and diabetes FSGS	[112]
m.5540G>A	MT-TW	Proteinuria Hypertension	Cataract	[113]
			Basal ganglia calcification	
			Retinitis pigmentosa	
m.9267G>C m.5913G>A	MT-CO3 MT-CO1	Hypertension	Mitochondrial diabetes DKD	[114]
		Nephropathy		
		Hyperglycemia		
		Insulin resistance		
		Deafness		
m.7501T>A	MT-tRNA^Ser^	Proteinuria	Glomerulosclerosis Diabetes mellitus	[115]
		Hypertension		
		Hyperglycemia		

Abbreviations: eGFR, estimated glomerular filtration rate; FSGS, focal segmental glomerulosclerosis; DKD, diabetic kidney disease; AKI, acute kidney injury; ChRCC, chromophobe renal cancer; CKD, chronic kidney disease.

**Table 2:** Therapeutic intervention for mtDNA in different kidney models.

Therapeutic Interventions	Models	Main Effects on mtDNA and Mitochondrial Function	Reference
Fluorofenidone	UUO and IRI	Increased mtDNA copy number Increased TFAM and PGC-1α expression Maintained mitochondrial structure Reduced mitochondrial oxidative stress	[90]
l-carnitine	DKD	Decreased circulating mtDNA content Reduced mtROS production Suppressed inflammation	[124]
Sacubitril/valsartan	DKD	Albuminuria Inhibited cGAS-STING signaling Decreased oxidative response	[126]
Coenzyme Q_10_	IRI	Alleviated mtDNA damage Suppressed inflammatory and oxidative responses	[127]
Treprostinil	IRI	Increased mtDNA copy number Increased PGC-1α expression	[128]
		Increased ATP level	
		Reduced mitochondrial oxidative injury	
Roxadustat	IRI	Increased ATPβ and PPARγ expression, and mtDNA	[129]
		Alleviated DNA damage	
Celastrol	Cisplatin-induced AKI	Increased mtDNA copy number Increased MMP Restored OXPHOS activity	[130]
H151	Cisplatin-induced AKI	Restored mtDNA content Reversed mitochondrial gene expression Suppressed inflammation	[131]
Adiponectin	DKD	Increased mtDNA content Increased TFAM and PGC-1α expression Increased mitochondrial mass Increased MMP	[132]
Salidroside	DKD	Increased mtDNA copy number Enhanced ETC proteins Increased PGC-1α expression	[133]
Artemether	Adriamycin nephropathy	Restored redox imbalance Increased mtDNA copy number	[134]
		Improved mitochondrial function	

Abbreviations: UUO, unilateral ureteral obstruction; IRI, ischemia-reperfusion injury; PGC-1α, peroxisome proliferator-activated receptor gamma coactivator-1α; ATPβ, ATPase β subunit; PPARγ, peroxisome proliferator-activated receptor γ; MMP, mitochondrial membrane potential; DKD, diabetic kidney disease; mtROS, mitochondrial reactive oxygen species; cGAS, cyclic GMP-AMP synthase; STING, stimulator of interferon genes.

## Data Availability

Not applicable.
